# Danish translation and linguistic validation of the BODY-Q: a description of the process

**DOI:** 10.1007/s00238-016-1247-x

**Published:** 2016-10-08

**Authors:** Lotte Poulsen, Michael Rose, Anne Klassen, Kirsten K. Roessler, Jens Ahm Sørensen

**Affiliations:** 1 Department of Plastic Surgery, Odense University Hospital, Sdr. Boulevard 29, 5000 Odense, Denmark; 2 Department of Plastic Surgery, Hospital of Southwest Jutland, Esbjerg, Denmark; 3 Department of Pediatrics, McMaster University, Hamilton, Canada; 4 Department of Psychology, University of Southern Denmark, Odense, Denmark; 5 Department of Plastic Surgery, Odense University Hospital, Odense, Denmark

**Keywords:** Translation, Cultural adaption, Linguistic validation, Patient-reported outcome, Bariatric surgery, Body contouring surgery

## Abstract

**Background:**

Patient-reported outcome (PRO) instruments are increasingly being included in research and clinical practice to assess the patient point of view. Bariatric and body contouring surgery has the potential to improve or restore a patient’s body image and health-related quality of life (HR-QOL). A new PRO instrument, called the BODY-Q, has recently been developed specifically for this patient group. The aim of the current study was to translate and perform a linguistic validation of the BODY-Q for use in Danish bariatric and body contouring patients.

**Methods:**

The translation was performed in accordance with the International Society For Pharmacoeconomics and Outcomes Research (ISPOR) and the World Health Organization (WHO) recommendations. Main steps taken included forward and backward translations, an expert panel meeting, and cognitive patient interviews. All translators aimed to conduct a conceptual translation rather than a literal translation and used a simple and clear formulation to create a translation understandable for all patients.

**Results:**

The linguistic translation process led to a conceptually equivalent Danish version of the BODY-Q. The comparison between the back translation of the first Danish version and the original English version of the BODY-Q identified 18 items or instructions requiring re-translation. The expert panel helped to identify and resolve inadequate expressions and concepts of the translation. The panel identified 31 items or instructions that needed to be changed, while the cognitive interviews led to seven major revisions.

**Conclusions:**

The impact of weight loss methods such as bariatric surgery and body contouring surgery on patients’ HR-QOL would benefit from input from the patient perspective. A thorough translation and linguistic validation must be considered an essential step when implementing a PRO instrument to another language and/or culture. A combination of the ISPOR and WHO guidelines contributed to a straightforward and thorough translation methodology well suited for a Danish translation of the BODY-Q. The described method of translation and linguistic validation can be recommended for future translations of PRO instruments in the field of plastic surgery.

Level of Evidence: Not ratable.

## Introduction

In recent years, there has been growing attention in health care concerning the evaluation of outcomes from the patient perspective. Well-developed, psychometrically sound, and clinically meaningful patient-reported outcome (PRO) instruments are increasingly being included in research and clinical practice to assess the patient point of view. Conventional methods to assess outcomes, such as mortality data, complications data, and photo review represent the health care provider perspective. Earlier reviews of PRO instruments for bariatric and/or body contouring surgery have called for the development of a new and comprehensive PRO instrument [1–5]. An international team recently followed internationally accepted guidelines and methods for the development of a new PRO instrument specific to measuring outcomes in weight loss and body contouring [6–11]. This new PRO instrument, called the BODY-Q, includes a set of 18 independently functioning scales and an obesity-specific symptom checklist. The scales measure three main concepts: appearance, health-related quality of life (HR-QOL), and experience of care [12, 13].

When questionnaires are adapted to another language and culture, it is extremely important to perform a proper translation and linguistic validation of the instrument. Well-developed PRO instruments achieve content validity through careful qualitative interviews, which only increases the importance of performing a careful translation. The International Society For Pharmacoeconomics and Outcomes Research (ISPOR) and the World Health Organization (WHO) have developed guidelines for good practice in the translation, linguistic validation, and cultural adaption process [14, 15]. Following translation and linguistic validation for Danish patients, the BODY-Q could be used to support patient advocacy, patient education (e.g., satisfaction with information), and research efforts. Furthermore, using the BODY-Q scales in clinical practice will give patients the opportunity to report their concerns to their health care provider, who can use patients’ answers in clinical decision-making [16]. Thus, the aim of the current study was to translate and perform a linguistic validation of the BODY-Q in Danish bariatric and body contouring patients.

Bariatric surgery, in combination with body contouring surgery, aims to improve or restore a patient’s body image and HR-QOL. There have been many suggestions as to what constitutes the most important health concerns of the bariatric and body contouring surgery patient population, but little consensus on which questionnaires should be used to address these concerns [1–5]. A standardized approach to outcome assessment is needed, where only the most scientifically and clinically meaningful PRO instruments are used. Such an approach would advance knowledge about the impact of bariatric and body contouring surgery on patients and facilitate the ability to compare findings across studies and countries.

The BODY-Q [12, 13] addresses the need for a PRO instrument for weight loss and body contouring surgery patients [17, 18]. The BODY-Q was developed following internationally recommended guidelines for item generation, item reduction, and psychometric evaluation [6–11]. The content for the BODY-Q was developed from a literature review, 63 qualitative patient interviews, 22 cognitive patient interviews and input from 9 experts. The BODY-Q measures three domains (appearance, HR-QOL, and experience of health care) via 18 independently functioning scales and an obesity-specific symptom checklist. The extensive qualitative steps were used to ensure that the scales are clinically grounded, relevant, and meaningful and capture the patient perspective [12, 13]. The BODY-Q is unique from other PRO instruments in that it includes 10 scales to measure appearance-related concerns. These scales were created because appearance was found to be an important concern to patients undergoing weight loss and body contouring. In addition, concepts measured by the HR-QOL scales (body image and physical, psychological, social, and sexual functions) ask patients to answer “with their body in mind,” ensuring the data are condition-specific. Each BODY-Q scale is independently functioning (no total scores) and scored from 0 (worse) to 100 (best) score. The BODY-Q can be used to monitor patients over their entire weight loss journey. Use of a modern psychometric method (i.e. Rasch [19]) means that the BODY-Q scales are well suited for use in both research and clinical practice. Rasch analysis allows for more accurate measurement, thus improving the BODY-Q’s ability to measure clinically meaningful change compared to previously suggested instruments [12, 13, 20].

The aim of this study was to perform a Danish translation of The BODY-Q. Linguistic validation is the process to ensure that PRO concepts are equivalent and easily understood by people in countries not involved in the development. The translation was performed in accordance with the translation guidelines of ISPOR [14] and WHO [15]. The ISPOR guidelines describe principles of good practice for the translation and cultural adaptation process of PRO instruments and are, therefore, well suited for use in translating the BODY-Q. The WHO provides a framework with four steps i.e., forward translation, expert panel back translation, pre-testing, and cognitive interviewing and final version. The ISPOR guidelines define more refined steps i.e., preparation, forward translation, reconciliation, back translation, back translation review, harmonization, cognitive debriefing, review of cognitive debriefing results and finalization, proofreading, and final report.

## Material and methods

We obtained permission to use the BODY-Q from the developers (Klassen et al., 2014). Ethical approval was applied from the Danish Ethics board prior to beginning the study. The translation process is described in Table 1. To begin, the project coordinators developed explanations for concepts measured by the BODY-Q and translators and expert panel participants were recruited. All translators aimed to create a conceptual translation rather than a literal translation and used a simple and clear formulation to create a translation that was understandable for all patients. The following six steps were taken:

**Table 1:** Overview of the translation process and adopted steps

Step	ISPOR guidelines	WHO guidelines	Our study
1	Preparation: Initial work before the translation begins as obtaining permission, inviting developers to participate, developing explanations for concepts in the instrument, and recruiting key persons.		Permission from the authors and agreement of participation. Explanations for concepts were developed, and translators plus expert panel participants were recruited.
2	Forward translation: Development of at least two independent forward translations and provision of explanation of concepts in the instrument to the key in-country persons and forward translators.	Forward translation: One translator, preferably a health professional, familiar with terminology of the area covered by the instrument and with interview skills should be given this task. The translator should be knowledgeable of the English-speaking culture but with mother tongue of the primary language of the target culture. The translation is recommended to be conceptual rather than literal, and the language should be natural and acceptable for the broadest audience.	We performed two forward translations. One was performed by a professional translator, and one was performed by a clinician with experience with the patient population, both with Danish mother tongue and fluent in English. A conceptual instead of literal translation was developed.
3	Reconciliation: Reconciliation of the forward translations into a single forward translation.		Reconciliation and harmonization meeting leading to agreement on Danish version 1.
4	Back translation: Back translation of the reconciled translation into the source language.	Back translation: The instrument should be translated back to English by an independent translator, whose mother tongue is English and who has no knowledge of the questionnaire. Back translation will be limited to selected items. As in the initial translation, emphasis in the back translation should be on conceptual and cultural equivalence and not linguistic equivalence. Discrepancies should be discussed with the editor-in-chief and further work (forward translations, discussion by the bilingual expert panel, etc.) should be iterated as many times as needed until a satisfactory version is reached.	Professional independent backward translation (mother tongue English and fluent in Danish). A conceptual instead of literal translation was aimed.
5	Back translation review: Review of the back translations against the source language.		Comparison of the original BODY-Q and the back translated Danish version with the authors (Dr. Klassen and Dr. Pusic). The differences were discussed, and the process was repeated until a satisfactory result was achieved, leading to Danish version 2.
6	Harmonization: Harmonization of all new translations with each other and the source version.	Expert panel: The expert panel should aim to identify and resolve inadequate expressions and concepts of the translation, as well as any discrepancies. The panel is recommended to be bilingual and should include translators, experts in health, as well as experts with experience in instrument development and translation.	Expert panel meeting with the participation of all translators, a specialist in bariatric surgery and a specialist in body contouring surgery, leading to consensus on Danish version 3 for pre-testing/cognitive debriefing. All participants were fluent in both Danish and English. All documents and guidelines were presented to all participants prior to the meeting.
7	Cognitive debriefing: Cognitive debriefing of the new translation, usually with patients drawn from the target population.	Pre-testing and cognitive interviewing: Pre-test respondents should include individual representative of those who will be administered the questionnaire. A number of minimum 10 for each section are recommended, and they should represent a variation of the target population. Pre-test respondents should be systematically debriefed and asked thorough concerning understandability and alternative wording.	Cognitive patient interviews (six pre-bariatric surgery [one patient was dyslectic], five pre-body contouring surgery, and five post-body contouring surgery). Further cognitive patient interviews (two pre-bariatric surgeries, two pre-body contouring surgeries, and two post-body contouring surgeries). All together, we performed 22 cognitive interviews.
8	Review of cognitive debriefing results and finalization: Cognitive debriefing results are reviewed and the translation finalized.		Reconciliation and harmonization leading to Danish version 4.
9	Proofreading: The finalized translation is proofread.	Final version: The final version of the instrument in the target language should be the result of all the iterations described.	Finalization and proofreading leading to the final Danish version of the BODY-Q.
10	Final report: Report is written on the development of the translation.	Documentation: All steps should be traceable through appropriate documents including the different translations and a description of changes made as result of the expert panel meeting and the cognitive debriefing.	A final report was written on the translation process for documentation. The report includes all relevant documents.

1. Two independent forward translations were performed. A professional translator performed one, and a clinician with experience with the patient population performed the other. Both forward translators had Danish as their mother tongue and were fluent in English. A harmonization meeting between the two forward translators was held in order to achieve agreement on Danish version 1.

2. An independent professional translator produced a backward translation of the harmonized version. The translator had English as his mother tongue and was fluent in Danish. The back-translated version was compared with the original BODY-Q. All discrepancies were noted and discussed with the BODY-Q developers (Drs. Klassen and Pusic). Items from the back translation with different meaning than the English version were re-translated and shown to the developers. This process continued until a satisfactory result was achieved, leading to Danish version 2.

3. The translation team hosted an expert panel meeting. Prior to the meeting, the Danish version of the BODY-Q, along with translation guidelines, was sent to participants to review. The three translators, a specialist in bariatric surgery, and a specialist in body contouring surgery attended the meeting. All participants had Danish as their mother tongue and were fluent in English, except the back translator who had English as his mother tongue and was fluent in Danish. The aim of the meeting was to determine if the Danish version of the BODY-Q was understandable and measured all clinically relevant issues from the perspective of the clinicians. Feedback received was used to revise the scales, leading to consensus on Danish version 3 for pre-testing.

4. In the fourth step, 16 cognitive interviews were conducted with patients to determine if the BODY-Q instructions, response options, and items were clear, unambiguous, and relevant to respondents. Participants included six pre-bariatric (one patient was dyslectic), five pre-body contouring, and five post-body contouring. Patients were asked to read through the BODY-Q and discuss how they understood each item and the associated response options. Findings were used to make further adjustments to the translation, leading to Danish version 4.

5. In the fifth step, we conducted a further six cognitive interviews with patients with Danish version 4. Participants included two pre-bariatric, two pre-body contouring, and two post-body contouring. Findings were used to make further adjustments leading to Danish version 5.

6. In the last step, Danish version 5 was proofread independently by two clinicians, leading to the final Danish version of the BODY-Q.

## Results

The translation process led to a Danish version of the BODY-Q that was linguistically validated and conceptually equivalent to the original English version. An example of the major changes made throughout the translation process for one BODY-Q scale can be found in the Appendix.

In step 1, we found that the two independent forward translations had different views on the language, which required discussion in order to reach consensus. For some items, the clinician had included medical terms that the other translator thought would be challenging for patients to understand. However, the wordings of some items translated by the professional translator were judged to reflect insufficient knowledge of the patient group and journey. During the reconciliation and harmonization meeting, the two perspectives were found to provide complimentary information. Following discussion and revision, the two translators reached consensus on the Danish version 1.

The comparison of the back translation of Danish version 1 and the original English version with the developers of the BODY-Q identified 18 items or instructions where the meaning differed, requiring re-translation of the items and review by the developers. This iterative process was instrumental in helping to secure a conceptual as opposed to literal translation. For example, in the scale that measures body image, the original item “I feel positive towards my body” was initially back translated as “I have a positive relationship with my body,” which was judged to have a different meaning and, thus, required re-translation. Another example was the use of the word “bothered” in the instructions of the scales measuring appearance of body contouring scars and excess skin. The word “bothered” proved difficult to translate into Danish, a challenge that has also previously been described [21]. The interactive process of comparing the Danish and English versions in an ongoing discussion with the developers helped to find consistent conceptual solutions.

The expert panel helped to identify and resolve unsatisfactory expressions and concepts in the back translation. Specifically, 31 items or instructions needed to be changed. For example, in the original version of the BODY-Q, the instructions for the patient experience scales that measure satisfaction with medical team included psychologists as a member of the medical team. Since psychologists are not part of medical teams for bariatric and/or body contouring care in Denmark, the expert panel decided to remove the word psychologist from the instructions. The expert panel meeting resulted in consensus on the Danish version 3 for cognitive debriefing.

Table 2 shows the characteristics of the participants included in the cognitive interviews. Patients were selected to ensure representation from all phases of the patient journey. Patients were debriefed about the study and asked to read systematically through the BODY-Q to identify problems (e.g. awkward or ambiguous wording) with the instructions, response options, and items and to suggest potential alternative wording. For example, some male patients pointed out that the Danish translation of “swimsuit” in the scales measuring the appearance of the abdomen and the body overall made them think of a female swimsuit. This finding was used to change the word for swimsuit to a gender-neutral word that retained the same meaning. The first set of 16 interviews provided high-quality input that led to seven major revisions, reconciliation, and harmonization (Danish version 4). The revised version, shown to six more participants, led to only minor changes. These changes were discussed followed by reconciliation and harmonization leading to the Danish version 5. Patient feedback was overwhelmingly positive. Patients found the scales to be relevant and easily understandable. Some patients even expressed that it put words to feelings and thoughts that they had not been able to previously express. The final proofreading in step 6 led to minor changes in grammar, resulting in the final linguistically validated and equivalent Danish version of the BODY-Q.

**Table 2:** Patient characteristics; cognitive interviews

First round of interviews	
Patients pre-bariatric surgery (*n* = 6)	
Female	4
Male	2
Age	40 (30–51)
BMI	44, 04 (36–51)
Patients pre-body contouring surgery (*n* = 5)	
Female	5
Male	0
Age	46, 6 (32–56)
BMI	26, 98 (22–32)
Patients post-body contouring surgery (*n* = 5)	
Female	4
Male	1
Age	41, 6 (34–53)
BMI	27, 95(24–36)
Second round of interviews	
Patients pre-bariatric surgery (*n* = 2)	
Female	1
Male	1
Age	43 (26–60)
BMI	44, 89 (41–49)
Patients pre-body contouring surgery (*n* = 2)	
Female	2
Male	0
Age	34, 5 (28–41)
BMI	25, 25 (25–26)
Patients post-body contouring surgery (*n* = 2)	
Female	2
Male	0
Age	45 (43–47)
BMI	24, 73 (23–26)

## Discussion

The focus on PRO is increasing, and there is no doubt that the ongoing discussion of the impact of weight loss methods, such as bariatric surgery and/or body contouring surgery, on patients’ HR-QOL would benefit from input from the patient perspective. In Denmark, both bariatric and body contouring surgeries are performed in the public health care system [22] and there is an increasing economic focus on the importance of being able to show benefits of the treatment provided. Since 2010, all patients undergoing bariatric surgery have been registered in the Danish Bariatric Surgery Database [23]. The database has eight indicators, including complications data, weight loss data, and effect on comorbidity. This database (for both bariatric and body contouring patients) also measures HR-QOL using the Moorehead-Ardelt Quality of Life Questionnaire (MAQOL) [24]. The MAQOL is a 6-item scale that asks about self-esteem, physical, social, work, sexual, and eating behavior. This PRO instrument has important limitations in terms of content validity when used in body contouring patients, i.e., it does not ask about “appearance” even though weight loss following bariatric surgery can result in excess hanging skin that has a negative impact on body image and HR-QOL. Other important limitations of the MAQOL include a total score summing the six items, which has an ambiguous meaning; patients were not involved in its development (no qualitative interviews) [5–10]; and the scale was not designed to measure change across the entire weight loss journey. These limitations make the MAQOL an inappropriate tool to use as an indicator for quality monitoring and usefulness in the individual patient journey. Nonetheless, perhaps due to a lack of an appropriate PRO instrument, the MAQOL has been implemented in the Danish national database for bariatric and body contouring patients. The BODY-Q [12, 13] provides a comprehensive set of scales that could now be applied in the Danish bariatric and body contouring surgery patients. Strengths of the BODY-Q include that it was developed according to recommended guidelines for PRO instrument development [6–11], and it is clinical grounded, addressing relevant concerns of patients, including appearance concerns, HR-QOL, and experience of care. In the UK, the National Health Service (NHS) has recently recommend the use of select BODY-Q scales with all patients undergoing liposuction and abdominoplasty [25].

The ISPOR and WHO guidelines [14, 15] were adopted for the translation process, and a culturally adapted and equivalent Danish version of the BODY-Q was achieved. Using a combination of the two guidelines, we found the translation methodology to be straightforward, thorough and well suited for Danish translation of the BODY-Q. As previously described, there are a few differences between the ISPOR and WHO guidelines and both have strengths and limitations. For example, the ISPOR guidelines [14] recommend that two translators perform forward translation independently followed by a reconciliation meeting, whereas the WHO guidelines [15] emphasize the importance of achieving a conceptual rather than literal translation through the forward and back translations. The WHO guidelines explicitly recommend an expert panel. This step proved to be important in the translation of the BODY-Q into Danish and led to several crucial changes in both the wording of instructions and items. One could argue that we should also have included patients in the expert panel, but instead we conducted a large number of cognitive interviews and found that this approach was an acceptable way of ensuring that the patient voice was well represented. Furthermore, the WHO guideline includes a greater focus on the cognitive debriefing stage, which we also found to be of extreme importance. Feedback from patients was crucial and led to linguistic changes that improved the acceptability of the final scales. The strength of our cognitive interviewing was the number of patients we were able to include, as well as the ability to hand pick patients to ensure that different points on the weight loss journey were represented. A limitation in our sample was the smaller number of men interviewed (overall 18 %) compared to women (overall 81 %). However, this difference reflects the distribution of gender in the bariatric and body contouring population [26].

Overall, we found that the combination of methods outlined by ISPOR and WHO provided a rigorous process that led to a high quality Danish translation of the BODY-Q. PRO instruments such as the BODY-Q are rapidly setting the standard for outcome measurement within the field of plastic and reconstructive surgery. The methods of translation and linguistic validation described here could be used to produce other translations of the BODY-Q, as well as other PRO instruments. Once a PRO instrument is rigorously translated and linguistically validated, it is important to ensure psychometric validation [20]. The next step of our research plan is to field test the BODY-Q in a large sample of bariatric and body contouring patients.

## Conclusion

The translation and validation processes are an essential step in adapting a PRO instrument to another language and/or culture. We have translated the BODY-Q into Danish and tested its cultural relevance in a group of Danish patients undergoing bariatric and body contouring surgery. We found the translation methodology to be straightforward. The expert panel meeting and the cognitive debriefing were particularly useful steps taken to create a culturally equivalent translation. A thorough translation and a linguistic validation are of great importance when implementing a PRO instrument, and the described method of translation and linguistic validation can be recommended for future translations of PRO instruments in the field of plastic surgery.

## Appendix

**Table 3:** Example of discrepancies and changes in the translation process

Name of scale	Forward translation	Backward translation	Changes after comparison of the original and back translated version	Expert panel meeting	Cognitive patient interviews	Further cognitive patient interviews
Appearance Body contouring scars						
These questions ask about your body contouring scars. For each question circle only one answer. With your body contouring scars in mind, in the past 2 weeks, how much have you been bothered by:	“Bothered” was a difficult word to translate into Danish.	These questions deal with your body contouring scars. Circle one answer for each question. With your body contouring scars in mind, how much have you during the last 2 weeks been troubled by:	The importance of securing a conceptual translation of “bothered” was highlighted. Remaining discrepancies were found with the same conceptual meaning.	It was ensured that the Danish translation for “bothered” was conceptually the same. Remaining discrepancies were also found to be with the same conceptual meaning.	Several patients post-body contouring revealed that they found “body contouring scars” strange and that they instead would suggest “surgical scars,” which make more sense in Danish. Patients explained that they found the Danish translation of “bothered” easily understandable and interviewing confirmed a correct conceptual translation.	No changes. Patients found “surgical scars” easily understandable and interviewing confirmed a correct conceptual translation.
1. Having to dress in a way to hide your scars?	In Danish, there are two very similar ways of saying “having to” with one being a bit more demanding than the other. The least demanding option was chosen to get as close to a conceptual translation as possible	That you must dress in a way that hides your scars?	No changes. “Having to” and “must” were found with the same conceptual meaning.	No changes	No changes. Patients explained that they found the item easily understandable and interviewing confirmed a correct conceptual translation.	No changes
2. How wide your scars look?	Straightforward	How wide your scars look?	No changes	No changes	No changes	No changes
3. Location of your scars?	Straightforward	The position of your scars?	No changes. “Location” and “position” were found with the same conceptual meaning.	No changes	No changes	No changes
4. The length of your scars?	Straightforward	The length of your scars?	No changes	No changes	No changes	No changes
5. How noticeable your scars are?	Straightforward	How visible your scars are?	The importance of securing a conceptual translation of “noticeable” was highlighted. “Noticeable” was not found to be with the same conceptual meaning.	“Visible” was changed, so that the conceptual meaning of “noticeable” was ensured.	No changes. Patients explained that they found the item easily understandable and interviewing confirmed a correct conceptual translation.	No changes
6. The color of your scars?	Straightforward	The color of your scars?	No changes	No changes	No changes	No changes
7. How thick your scars look (i.e., raised or bumpy)?	In Danish, the words “raised and bumpy” are seldom used about scars. The conceptual meaning was thought easily understandable, and it was straightforward to find Danish words to replace.	How thick (i.e., swollen or uneven) your scars look?	No changes. Together “swollen and uneven” was by the authors found to be conceptually equivalent with the original version.	The expert panel found a better Danish word for “raised” leading to improved conceptual translation.	No changes. Patients explained that they found the item easily understandable, and especially the translation for “raised or bumpy” in brackets confirmed the meaning.	No changes
8. Your scars looking crooked (i.e., not in a straight line)?	Straightforward	That your scars look crooked (i.e. not in a straight line)?	“Your scars looking crooked” and “That your scars look crooked” were defined as two different ways of expressing the same meaning.	No changes	No changes	No changes
9. People seeing your scars?	Straightforward	That people see your scars?	“People seeing” and “That people see” were defined as two different ways of expressing the same meaning.	No changes	No changes	No changes
10. How your scars look when they are not covered by clothes?	Straightforward	How your scars look when they are not covered by clothes?	No changes	No changes	No changes	No changes
